# Heat Stress Reduces Root Meristem Size via Induction of Plasmodesmal Callose Accumulation Inhibiting Phloem Unloading in *Arabidopsis*

**DOI:** 10.3390/ijms23042063

**Published:** 2022-02-13

**Authors:** Jie Liu, Yao Liu, Shuang Wang, Yongqi Cui, Dawei Yan

**Affiliations:** 1 State Key Laboratory of Crop Stress Adaptation and Improvement, School of Life Sciences, Henan University, Kaifeng 475001, China; liujie19900115@yeah.net (J.L.); m17633750636@163.com (Y.L.); ws2020210@163.com (S.W.); 17698011118@163.com (Y.C.); 2 Academy for Advanced Interdisciplinary Studies, Henan University, Kaifeng 475001, China

**Keywords:** callose, callose synthase, heat, root meristem, plasmodesmata, thermotolerance

## Abstract

The intercellular transport of sugars, nutrients, and small molecules is essential for plant growth, development, and adaptation to environmental changes. Various stresses are known to affect the cell-to-cell molecular trafficking modulated by plasmodesmal permeability. However, the mechanisms of plasmodesmata modification and molecules involved in the phloem unloading process under stress are still not well understood. Here, we show that heat stress reduces the root meristem size and inhibits phloem unloading by inducing callose accumulation at plasmodesmata that connect the sieve element and phloem pole pericycle. Furthermore, we identify the loss-of-function of *CALLOSE SYNTHASE 8* (*CalS8*), which is expressed specifically in the phloem pole pericycle, decreasing the plasmodesmal callose deposition at the interface between the sieve element and phloem pole pericycle and alleviating the suppression at root meristem size by heat stress. Our studies indicate the involvement of callose in the interaction between root meristem growth and heat stress and show that CalS8 negatively regulates the thermotolerance of *Arabidopsis* roots.

## 1. Introduction

Phloem-mediated translocation of molecules including nutrients, proteins, RNAs, and hormones is critical for plant growth and development [1,2,3,4,5,6]. The process by which solutes exit the phloem, referred to as phloem unloading, regulates the molecular flux into sink organs to accommodate their high energy and signaling demands [7]. Phloem unloading is mostly active in growing sink tissues such as the root apex, and the restriction of phloem unloading impairs primary root growth [7,8]. Several regulators of phloem unloading have been identified up to now [8,9,10,11,12]. For example, knocking out two annexin genes in *Arabidopsis*—namely, *ANNEXIN1* and *ANNEXIN2*—limited the unloading of sugars from the phloem to the root tip, and this restriction impaired primary root growth [8]. The *PHLOEM UNLOADING MODULATOR* gene regulates the early root elongation rate by controlling the extent of phloem unloading into the root meristem [9]. Plasmodesmata (PD)-mediated symplastic trafficking plays an important role in the unloading process [13]. PD are membrane-lined channels connecting adjacent cells, exhibiting diverse and dynamic morphology and inner structure at different cell layers and developmental stages of plants [14,15]. In *Arabidopsis*, solutes are batch-unloaded from protophloem sieve element (PSE) into the phloem-pole pericycle (PPP) facilitated by funnel-shaped PD [16], and subsequently move further into the endodermis [9]. This post-SE unloading process is modulated by the dynamic proportion of type I and type II PD at the PPP–endodermis interface [9,17].

Callose (β-1,3-glucan) is known as a major regulator of plasmodesmal aperture and permeability, and its abundance is inversely associated with the trafficking efficiency through PD [18,19,20]. Dynamic callose turnover is achieved by the coordinated action of CalSs and β-1,3-glucanases (BGs) [21], and it regulates plant growth and development [10,11,22]. Gain-of-function mutations in *CalS3* lead to callose deposition at the PD and impair the cell–cell movement, resulting in the inhibition of primary root growth [11]. In *Arabidopsis*, β-1,3-glucanase_putative PD-associated protein (BG_ppap), BG1, BG2, and BG6 regulate callose degradation at the PD [22,23,24]. The plasmodesmal permeability also appears to be regulated by PD-LOCALIZED PROTEINS (PDLPs) and PLASMODESMATA CALLOSE-BINDING PROTEINS (PDCBs), of which the downstream targets are callose [25,26]. The mutants *pdlp5* and *pdcb1* showed increased PD permeability, whereas in the overexpression lines the intercellular trafficking was dramatically blocked [25,26]. Moreover, callose allows plants to rapidly coordinate symplastic signaling in response to abiotic stresses [20]. Chilling [27,28,29], wounding [30,31], and heavy metals [32,33,34] increase callose deposition at PD and inhibit root growth [24,32,35,36]. Given the rise in global temperature, heat has become one of the major stresses for plants [37]. Heat stress disturbs cellular homeostasis and impedes growth and development in plants [38]. In addition to the membrane damage, protein degradation, enzyme inactivation, and reactive oxygen species (ROS) accumulation, callose biosynthesis can also be induced by heat stress, even mild treatment [39,40,41]. However, very little is known about the mechanisms by which heat stress induces callose deposition. 

The *Arabidopsis* genome encodes 12 CalS proteins, and some of them show PD localization and specific expression patterns [11,16,21]. An inducible *cals3m* system under the *CalS8* promoter (*pCalS8::icals3m*) promotes callose accumulation in the PPP cells and reduces the PD permeability, leading to the blockage of symplastic PSE–PPP connection and the subsequent impaired phloem unloading and root growth [16]. CalS8 has been recognized as a key callose synthase modulating plasmodesmata-mediated trafficking in response to ROS-associated stress, since its absence arrests the induction of callose in response to H_2_O_2_ treatment and wounding [31]. These results confirm the inducible activity of CalS8 and imply the reasonable speculation of the possible role of CalS8 in modulating phloem unloading. In this study, we aimed to investigate whether heat stress affects root meristem size by inducing callose biosynthesis and which callose synthase is involved in this process. We found heat stress induces callose deposition at SE–PPP and PPP–endodermis interfaces, inhibits phloem unloading, and restricts the meristem size in *Arabidopsis* roots. Furthermore, we proved the loss of *CalS8* rescue the inhibition phenotype under heat stress due to the defect in callose accumulation around PD at the SE–PPP interface within the unloading zone of the root tip. Our study provides evidence suggesting *CalS8* acts as a negative modulator of plasmodesmata-mediated trafficking in response to heat stress in the root.

## 2. Results

### 2.1. Heat Stress Reduces Root Meristem Size and Inhibits Phloem Unloading

**Figure 1:**
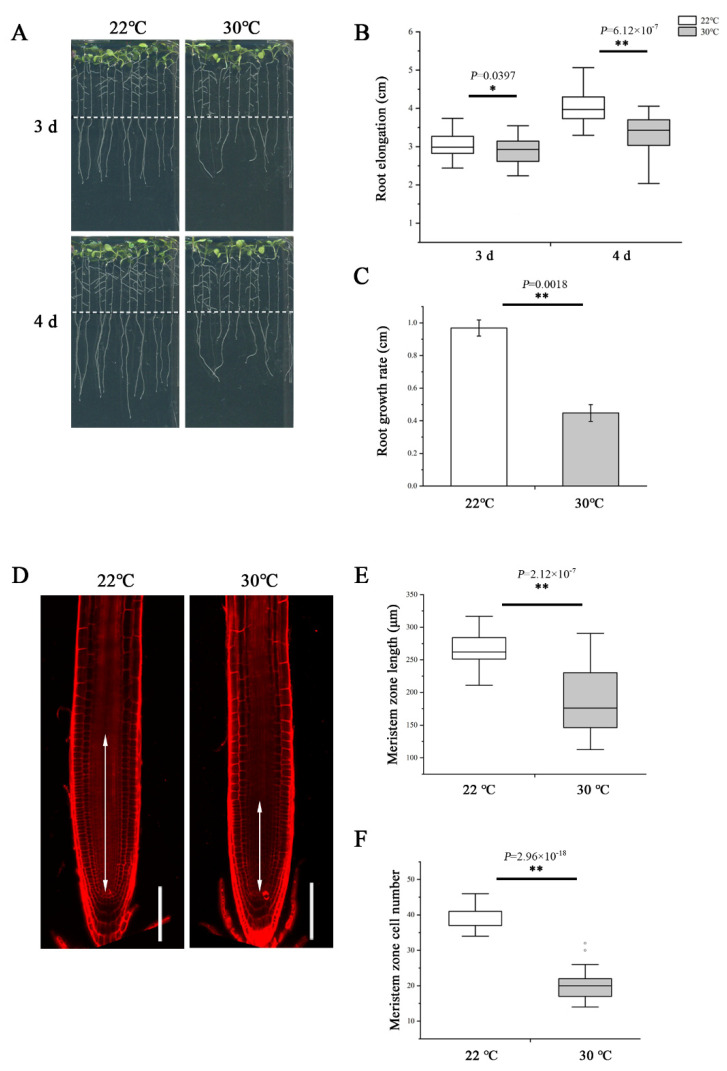
Heat stress reduces root meristem size in *Arabidopsis thaliana*. (**A**) Five-day-old wild-type (WT) seedlings grown on 1/2 MS medium were transferred to 22 °C and 30 °C and cultivated vertically for 3 d and 4 d and photographed. Dashed lines show the initial positions of root tips after being transferred. (**B**) Quantification of the length of newly grown primary roots in A, *n* >  34. (**C**) Quantification of the growth rate in A. The data are means ± SE. (**D**) Five-day-old roots of WT were transferred to 22 °C and 30 °C for 48 h and stained with propidium iodide to visualize the cell walls. Double arrows indicate the meristem length (from the cortical initial cell to the first elongated cell in the cortex layer). Scale bars = 100 μm. (**E**) Quantification of the root meristem length in D, *n* > 20. (**F**) Quantification of the cell number of meristem in D, *n* > 20. In the box plots (**B**,**E**,**F**), the boxes indicate the first and third quartiles, and the whiskers indicate the minimum and maximum values. The black lines within the boxes indicate the median values. Outliers are shown as dots. ** *p* < 0.01; * *p* < 0.05 (two-tailed, two-sample *t*-test).

To examine the impact of heat stress on primary root growth, we transferred 5-day-old wild-type *Arabidopsis thaliana* (Col-0) seedlings germinated and grown on normal 1/2 MS medium at 22 °C to 30 °C to grow continually. After 3- and 4-day treatment, we measured the elongation of newly grown primary roots and found the length was significantly shorter at 30 °C compared with 22 °C (Figure 1A,B). Similarly, the growth rate was also slower at 30 °C (Figure 1C). To assess the effect of high temperature in detail, we further examined the root meristem under both temperatures. Consistent with the change in root elongation, plants growing at 30 °C showed an evident reduction in the meristem size and cell number (Figure 1D–F). 

**Figure 2:**
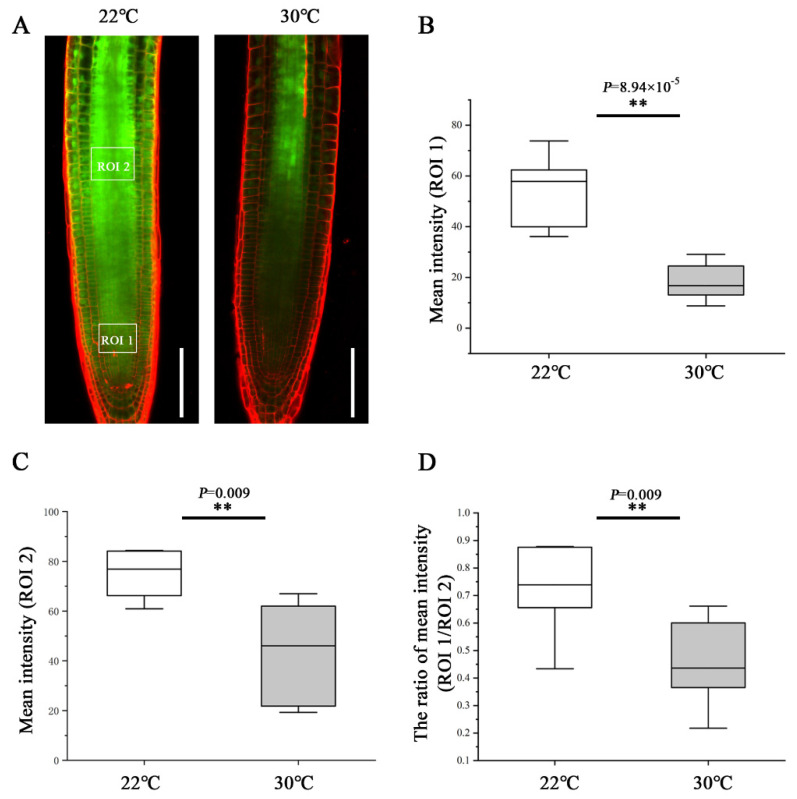
Heat stress inhibits phloem unloading in the root tips. (**A**) Comparison of the phloem unloading in the root of pSUC2::GFP-expressing 5-day-old seedlings at 48 h after being transferred to 22 °C and 30 °C. Scale bars = 100 μm. (**B**) Quantification of fluorescence intensity in an area of 50 × 40 µm^2^ (ROI1, 50 µm away from quiescent center (QC) in the central root meristem). (**C**) Quantification of fluorescence intensity in an area of 50 × 40 µm^2^ (ROI2, 300 µm away from QC in the stele). (**D**) Quantification the unloading capability from the phloem into the meristem by the ratio of GFP fluorescence intensity of an ROI1 relative to an ROI2. In the box plots (**B**–**D**), the boxes indicate the first and third quartiles, and the whiskers indicate the minimum and maximum values. The black lines within the boxes indicate the median values. ** *p* < 0.01 (two-tailed, two-sample *t*-test).

To determine the effect of heat stress on phloem unloading, we analyzed the phloem unloading using the transgenic plants expressing SUC2::GFP as mobile marker. GFP was expressed in companion cells and then transported and unloaded from the SE into PPP and diffused further via PD throughout the entire root meristem [10,16]. At 48 h after transfer to 30 °C, the GFP fluorescence intensity of the region of interest (ROI)1 in the root tip was much weaker than that of the roots still growing at 22 °C (Figure 2A,B). To quantify the unloading capability from the phloem into the meristem, we calculated the ratio of GFP fluorescence intensity of an ROI1 relative to an ROI2. Under the control condition of 22 °C, approximately 73% GFP was moved from the phloem to the meristem (Figure 2D). Whereas at 30 °C, the GFP unloading ratio was inhibited dramatically and decreased to 45% (Figure 2D). These results suggest that heat stress impedes the phloem unloading and limits the molecular flux to the root meristem, conferring the inhibition of primary root growth. 

### 2.2. Heat Stress Induces Plasmodesmal Callose Accumulation within Unloading Zone

**Figure 3:**
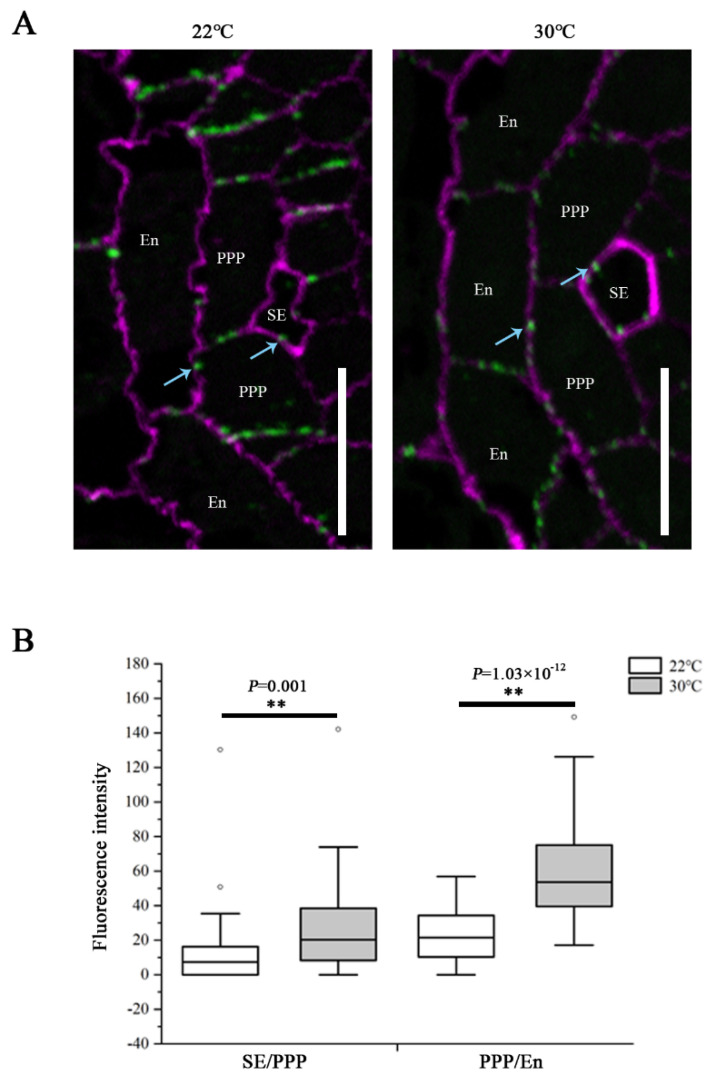
Heat stress induces callose deposition at SE–PPP and PPP–endodermal interfaces within the unloading zone. (**A**) Immunolocalization of callose at plasmodesmata (blue arrows) at the SE–PPP and PPP–endodermis interfaces in the same region of the unloading domain (200 μm upward of QC) in the root tips of Col-0 at 24 h after transferred to 22 °C and 30 °C. Scale bars = 10 μm. (**B**) Mean fluorescence intensity of the callose signals are calculated in ImageJ. In the box plots, the boxes indicate the first and third quartiles, and the whiskers indicate the minimum and maximum values. The black lines within the boxes indicate the median values. Outliers are shown as dots. ** *p* < 0.01 (two-tailed, two-sample *t*-test).

It is known that PD-mediated symplastic movement regulates phloem unloading in the root, and the callose turnover controls the plasmodesmal permeability [13,16,20]. To determine the influence of heat on PD conductivity, callose levels at PD were assessed by immunolocalization using a callose antibody on root sections derived from the unloading domain. We examined the plasmodesmal callose signals at both the SE–PPP and PPP–endodermal interfaces. The root tips of Col-0 seedlings transferred to 30 °C for 24 h showed a significant increase in callose amount at both interfaces, compared with that growing at 22 °C (Figure 3). These results indicate that the restriction of GFP unloading into the root meristem in response to heat stress resulted from excessive callose deposition at PD within the unloading zone, and this overproduction affected both the SE and post-SE unloading.

### 2.3. CalS8 Regulates the Plasmodesmal Callose Deposition under Heat Stress

To identify the candidate genes contributing to the callose accumulation involved in the heat response, we focused on the 12 *Arabidopsis* callose synthases. Based on their expression patterns and our previous work, we selected *CalS8* and *CalS6* as candidates due to their enrichment in the PPP cells [16] (Supplementary Figure S1). In targeting these two genes, we screened potential transfer T-DNA insertion lines and identified one putative *cals6* mutant and two *cals8* mutant alleles: *cals6*, *cals8-1,* and *cals8-2* (Supplementary Figure S2A,C). The corresponding transcripts were knocked out in *cals8-1* and *cals6* (Supplementary Figure S2B,D), which were thus used for further studies.

**Figure 4:**
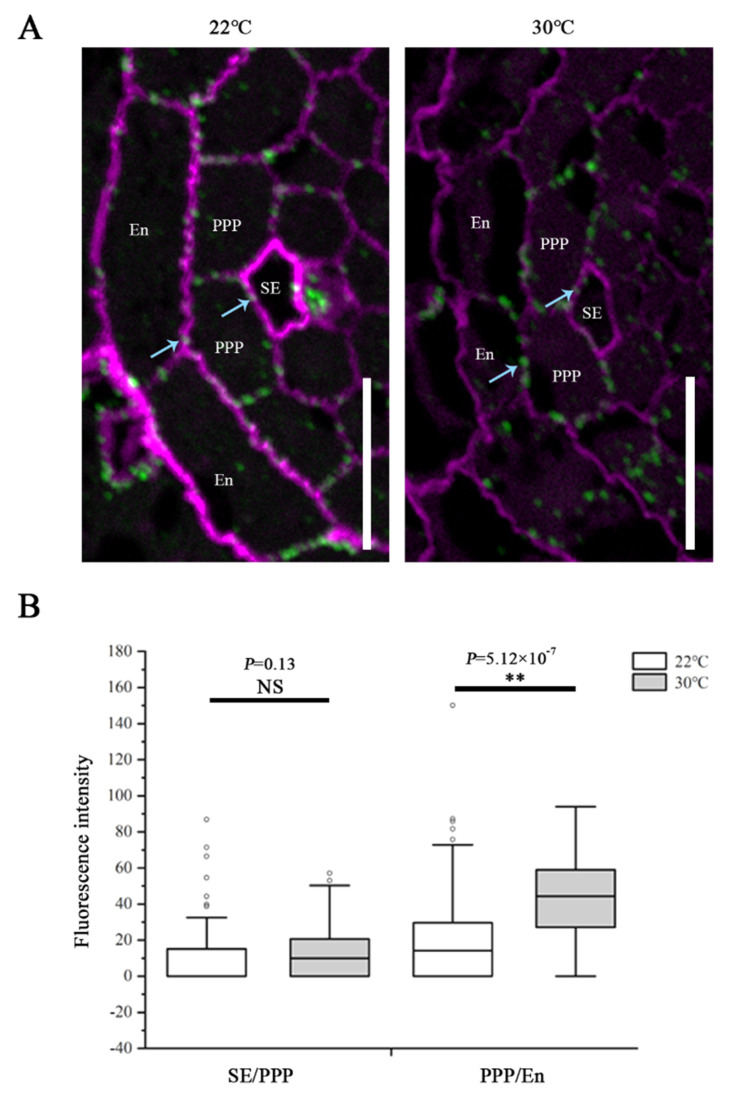
CalS8 is a bona fide callose synthase required for heat stress-induced callose deposition. (**A**) Immunolocalization of callose at plasmodesmata (blue arrows) at the SE–PPP and PPP–endodermis interfaces in the same region of the unloading domain (200 μm upward of QC) in the root tips of *cals8-1* at 24 h after transferred to 22 °C and 30 °C. Scale bars = 10 μm. (**B**) Mean fluorescence intensity of the callose signals are calculated in ImageJ. In the box plots, the boxes indicate the first and third quartiles, and the whiskers indicate the minimum and maximum values. The black lines within the boxes indicate the median values. Outliers are shown as dots. ** *p* < 0.01 (two-tailed, two-sample *t*-test). NS, not significant.

To determine the effects while *CalS8* and *CalS6* are absent, we examined the plasmodesmal callose levels in *cals8-1* and *cals6* mutants using the same method after the same heat treatment as above. Unlike the wild-type Col-0 plants, callose accumulation at the SE–PPP interfaces in the *cals8-1* roots were not increased when transferred to 30 °C from 22 °C. Conversely, callose is also increased significantly at the PPP–endodermal interface in *cals8-1* (Figure 4). These data suggest that *CalS8* is responsible for the plasmodesmal callose induction by heat stress at only the SE–PPP interfaces.

**Figure 5:**
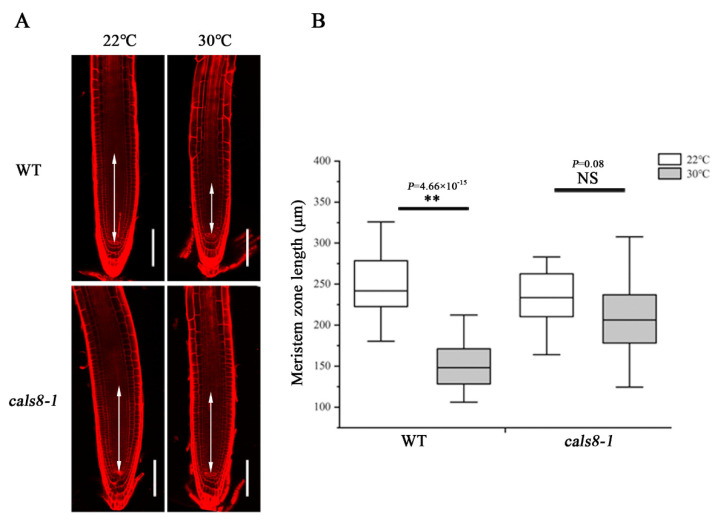
Loss of *CalS8* rescues the decrease in meristem size under heat stress. (**A**) Five-day-old roots of WT and *cals8-1* were transferred to 22 °C and 30 °C for 48 h and stained with propidium iodide to visualize the cell walls. Double arrows indicate the meristem zone. Scale bars = 100 μm. (**B**) Quantification of the meristem length in A, *n* > 20. In the box plots, the boxes indicate the first and third quartiles, and the whiskers indicate the minimum and maximum values. The black lines within the boxes indicate the median values. ** *p* < 0.01 (two-tailed, two-sample *t*-test). NS, not significant.

To test whether the reducing callose levels could ameliorate the inhibition of heat stress on primary root meristem growth, we compared the meristem length of *cals8-1* mutant and wild-type Col-0 roots after being transferred to 30 °C and 22 °C for 48 h. Unlike the decreased meristem size of Col-0 roots at 30 °C, there was almost no difference in *cals8-1* under these two conditions (Figure 5), indicating the enhanced thermotolerance of *cals8-1*. Nevertheless, this resistance was not exhibited in *cals6* mutant (Supplementary Figure S3). Overall, these results suggest a novel role of *CalS8* in the callose-mediated root meristem size inhibition under heat stress.

### 2.4. CalS8 Expression Is Not Influenced by Heat Stress

**Figure 6:**
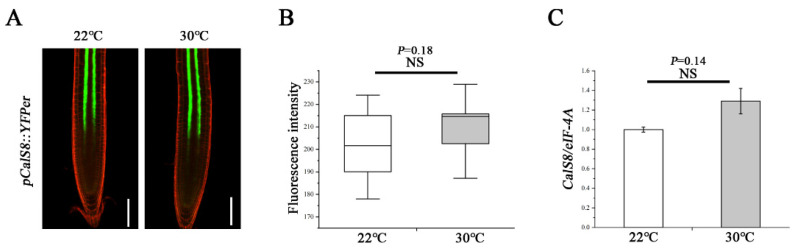
Transcript-level expression of *CalS8* is not influenced by heat stress. (**A**) Images of pCalS8::YFPer-expressing 5-day-old seedlings roots 24 h after transfer to 22 °C and 30 °C. Scale bars = 100 μm. (**B**) Quantification of fluorescence intensity in A using ImageJ. (**C**) Comparison of the relative transcript levels of *CalS8* in the root tips of Col-0 seedlings at 24 h after being transferred to 22 °C and 30 °C. Data are based on three independent replicate experiments. Data are means ± SD. In the box plots (**B**), the boxes indicate the first and third quartiles, and the whiskers indicate the minimum and maximum values. The black lines within the boxes indicate the median values. Significant differences were determined by the two-tailed, two-sample *t*-test. NS, not significant.

To explore how the heat regulates the *CalS8* function, we further tested whether the heat-induced callose deposition was accompanied by an elevated level of *CalS8* gene transcription. We first treated the transgenic plants harboring the *CalS8pro::YFPer* construct at 30 °C for 24 h. No significant difference in YFP reporter activity was observed at 30 °C and normal 22 °C (Figure 6A,B). We next verified this result by reverse transcription quantitative PCR (RT-qPCR). The expression level of *CalS8* were also hardly affected by heat stress (Figure 6C). Taken together, these data suggest that the callose induction at PD in response to the heat stress is not due to transcriptional activation of the *CalS8* gene. It remains to be tested whether this callose response is a result of post-transcriptional modification or activity promotion at the protein/enzyme level.

**Figure 7:**
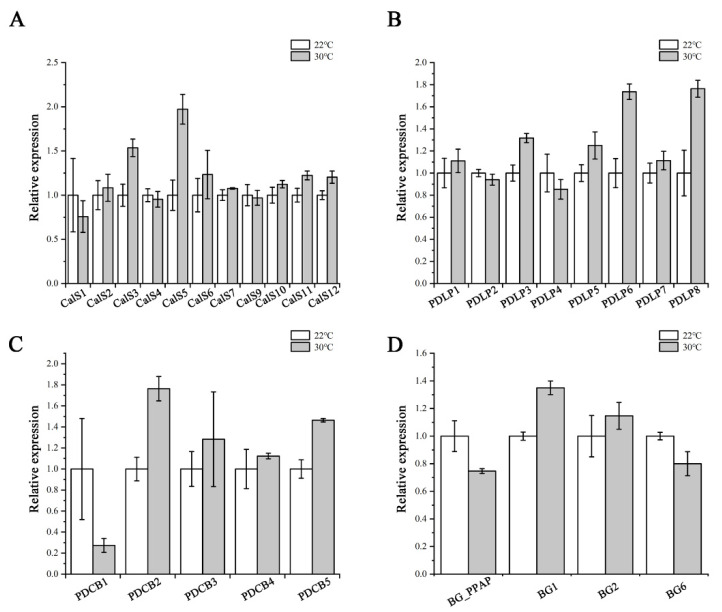
Expression of callose-associated genes after heat stress treatment. Quantitative RT-PCR analysis of *CalSs* (**A**), *PDLPs* (**B**), *PDCBs* (**C**), and *BGs* (**D**) transcript levels in root tips of Col-0 after 24 h heat stress treatment. Total RNA was isolated from root tips. *eIF-4A* was used as an internal control. Three independent experiments were performed. Data are means ± SE of three biological repetitions, *n* = 3.

Callose levels are regulated by the opposing activities of two enzyme families: callose synthases and β-1,3-glucanases, which produce and break down callose, respectively [21]. PDLPs and PDCBs are two key proteins families that positively regulate callose accumulation at PD [25,26]. We next used RT-qPCR to confirm the expression of PD callose associated genes, including the remaining eleven *CalSs*, four *β-1,3-glucanases* (*BG_PPAP*, *BG1*, *BG2*, *BG6*), eight *PDLPs,* and five *PDCBs*. As shown in Figure 7, no dramatic and significant upregulation in their expression levels was detected after heat treatment. This suggests that the heat stress probably does not interfere with callose biosynthesis via regulating most of the associated genes at the transcriptional level.

## 3. Discussion

Roots encounter various environmental challenges during development and respond by modulating their growth [42,43]. The root meristem is organized by stem cells that generate all cell types of the root [44]. The rates of cell production in the meristem and differentiation in the elongation/differentiation zone homeostatically maintain the meristem size. Under stresses, abnormal changes including deficient nutrient supply, altered hormone distribution, dysfunction of signaling, division/differentiation activities of transition zone, and meristematic zone regulate the root meristem size [24,42,43,45,46,47,48,49,50]. Heat stress impairs cell cycle, induces callose accumulation, impedes the carbon transport, and consequently inhibits the tissue growth [38,39,40,41,51]. Here, we find the mild heat stress (30 °C) reduces the root meristem size via callose-dependent suppression of source-to-sink phloem translocation. Our data uncover a new way of heat stress influencing root growth, while the mechanism of how the cell division is regulated during this process is worthy of further investigation.

Our current study provides evidence that *CalS8* contributes to the heat stress-induced plasmodesmal responses, but the upstream signaling components that coordinate plasmodesmal callose accumulation and permeability remain unclear. Several studies highlight a correlation between ROS levels and callose deposition at PD and the roles of ROS in the regulation of heat responses in plants [10,31,35,52]. *CalS8* has been characterized as a vital regulator mediating ROS-induced plasmodesmal callose deposition [31]. This raises the hypothesis that ROS might be an essential molecular link that integrates the heat stress with the restriction in plasmodesmal permeability caused by *CalS8*. The receptor-like proteins (RLPs) or receptor-like kinases (RLKs) are a highly expanded family of transmembrane proteins in plants and are largely responsible for communication between cells and the extracellular environmental stimuli [53]. The two extracellular DUF26 domains in RLPs and RLKs may be able to sense the apoplastic ROS status [54,55]. RLPs or RLKs, therefore, may be the candidates of the upstream regulators of CalS8 activity under heat stress, which needs more evidence.

Callose is one of the most active components of the cell wall due to its dynamic deposition and degradation. At plasmodesmata, the turnover of callose controls the plasmodesmal permeability and symplastic transport and consequently affects plant development and stress responses [10,11,18,22]. Our previous study indicated that phloem pole pericycle cells are the main mediator of unloading [9]. Overproduction of callose in the phloem pole pericycle compromises phloem unloading and root growth by blocking plasmodesmata [16]. We thus proposed that callose synthase that exhibits activity in the phloem pole pericycle might possess the ability of regulating phloem unloading. In this study, we found heat stress induces callose accumulation around plasmodesmata at both interfaces of sieve element–phloem pole pericycle and phloem pole pericycle–endodermis, and thus results in the inhibition of phloem unloading and root growth. Our data of root meristem analysis indicate that this inhibition by heat stress is probably initiated from a reduction in the meristematic cell proliferation. Nonetheless, this inhibition is suppressed by loss-of-function of *CalS8*, a callose synthase gene expressing specifically in the phloem pole pericycle. The root meristem grows almost normally, and the induction of callose deposition at plasmodesmata in the root unloading zone is reduced in *cals8-1* mutant under heat stress, suggesting a negative role of *CalS8* in thermotolerance. Unlike *CalS8*, the loss-of-function of *CalS6*, another callose synthase gene having similar expression pattern in phloem and phloem pole pericycle, does not show the rescued phenotype, indicating the functional divergence of callose synthases [21,56,57,58]. *CalS8* is previously proved to be involved in the basal and ROS-dependent plasmodesmal permeability in leaves [31]. In our study, we found the basal plasmodesmal callose levels at both the SE–PPP and PPP–endodermal interfaces were not altered in *cals8-1* mutant under normal growth temperature (Figure 3 and Figure 4), suggesting a potential functional redundancy with other *CalSs* in regulating plasmodesmal callose homeostasis for phloem unloading. Overall, these data indicate the possible specific role of *CalS8* in the heat-induced callose accumulation and root growth inhibition. Removal of *CalS8* is thus enhancing the thermotolerance of *Arabidopsis*, and similarly, it gives us a clue that investigating the homologous callose synthases that express in the phloem unloading zone and are induced by stress in crop plants would be of help for the improvement of stress-resistant species.

Based on the data of functioning in callose biosynthesis and plasmodesmata blockage [11,16,31], CalS8 is supposed to be localized at plasmodesmata or plasma membrane like other callose synthases [11]. Nevertheless, we failed to generate the stable clones of fluorescent protein-tagged CalS8 after many attempts, as well as did another research group [31]. Although the details of CalS8 localization remain unavailable, we found loss of *CalS8* arrests the induced plasmodesmal callose synthesis at the SE–PPP interface specifically but not the PPP–endodermis interface under heat stress. This suggests a possible biased localization of CalS8 at the SE–PPP interface or the existence of a compensation mechanism at the PPP–endodermis interface, which is worth investigating further. This might also imply the difference in functional regulation between SE–PPP and PPP–endodermis interfaces, although they are two connective key barriers for phloem unloading [9,13].

As with many other stresses, high temperature interferes with callose biosynthesis [59,60,61]. *CalS8* is involved in not only wounding-induced [31] but also heat-induced callose deposition and plasmodesmal regulation (Figure 4). Nonetheless, the transcript levels of *CalS8* were not altered significantly under both stress conditions [31] (Figure 6). We hence proposed such stresses might be able to regulate the upstream of *CalS8* or promote callose accumulation quickly via post-transcriptional modification or enzymatic activity regulation of CalS8 protein. Moreover, the expression levels of positive regulators of callose synthesis in our profile were all not increased dramatically under heat stress, with the fold changes less than 2 (Figure 7). Additionally, their expression patterns are not specific in phloem pole pericycle like *CalS8* [11,62,63]. We therefore propose that, given that the minor expression changes are imputable, they might contribute to the accumulation of callose in other tissues and other biological processes rather than the plasmodesmal callose impairing phloem unloading and root growth under heat stress.

## 4. Materials and Methods

### 4.1. Plant Materials and Growth

Arabidopsis ecotype Col-0 was used as the wild type. *cals8-1* (SALK_037603), *cals8-2* (SALK_076074), and *cals6* (SALK_022651C) were obtained from the Nottingham Arabidopsis Stock Centre (Nottingham, UK). The pSUC2::GFP lines were obtained from Ykä Helariutta’s lab (Cambridge, UK), and the pCalS8::YFPer line was obtained from Jung-Youn Lee’s lab (Newark, DE, USA). Seeds were sterilized in a solution containing 0.5% sodium hypochlorite and 0.1% Triton X-100 for 15 min, washed five times with sterilized water, and grown vertically in Petri dishes on 1/2 MS basal salt mixture with 0.7% Phytogel (Sigma-Aldrich, St. Louis, MO, USA), 1% sucrose, and 0.05% 4-morpholine ethanesulfonic acid (MES) at pH 5.8 in a growth chamber at 22 °C under 16 h light/8 h dark conditions.

### 4.2. Root Growth Analysis

For heat treatment, seeds were germinated vertically for 5 d at 22 °C before being transferred to 22 °C and 30 °C. The seedlings were photographed, and primary root length was measured 3 and 4 days after transfer. The meristem length and cell number were measured 48 h after transfer.

### 4.3. Callose Immunolocalization

Callose detection was conducted as described before [9,11]. Five-day-old wild-type and mutant seedlings were transferred to 22 °C and 30 °C for 24 h and then fixed in fixative solution containing 4% paraformaldehyde and 0.5% glutaraldehyde in 0.1 M PBS at pH 7.2 overnight at 4 °C. Roots were collected and washed with PBS and water and pre-embedded in low-melting agarose. The samples then were dehydrated with ethanol of different concentration gradients (25%, 50%, 70%, 90%, 96%, 3 × 100%) for 30 min each step. After resin infiltration by increasing resin concentrations (LR white medium grade, 33%, 66%, 3 × 100%), polymerization was conducted overnight at 60 °C. Selected the unloading domain where 200 μm upward of Quiescent center and performed 1 μm semi-thin sections on Leica EM UC7. Callose immunolocalization sections were incubated with diluted primary antibodies (monoclonal antibody against (1→3)-β-glucan (Biosupplies), 1:1000) and secondary antibodies (Alexa Fluor Plus 488 goat anti-mouse IgG, 1:1000). Slides were finally mounted in a 1:1 solution of AF1 antifading agent (Citifluor) and 1 × PBS, with the addition of calcofluor as a cell wall counterstain and scanned by confocal microscope.

### 4.4. Phloem Unloading Assay

Five-day-old seedlings expressing pSUC2::GFP were transferred to 22 °C and 30 °C for 48 h, respectively, and roots were imaged by confocal microscope for quantifying GFP fluorescence. Propidium iodide was used as a counter stain for the cell wall. Ratiometric data were analyzed by taking fluorescence intensity ratios in an ROI at 50 μm above the QC relative to an ROI at 300 μm away from QC in the stele in ImageJ.

### 4.5. Transcriptional Analyses

To quantify the expression levels of mRNA, root tips from 5-day-old seedlings were harvested after being transferred to 22 °C and 30 °C for 24 h. Total RNA was extracted by hot phenol method and then treated with RNase-free DNase I (Takara, Shiga, Japan) to remove genomic DNA. Complementary DNAs were synthesized from total RNA primed with oligo(dT)18 primers using M-MLV reverse transcriptase (Takara) according to the user’s manual. Semi-quantitative PCR for *cals8-1*, *cals8-2,* and *cals6* was performed using specific primers across the T-DNA insertion site and *eIF-4A* as an internal standard. Quantitative PCRs were performed using gene-specific primers and real-time PCR mix (Roche, Basel, Switzerland) in a LightCycler 480 Real-Time PCR System (Roche) with a standard program for 40 cycles. The levels of gene expression were calculated relative to *eIF-4A*. The primer sequences are listed in Supplementary Table S1.

### 4.6. Confocal Imaging

All confocal images were obtained by Nikon/A1 confocal laser scanning microscope. Images of callose immunolocalization were scanned under a water-immersion 60× objective at ×2 digital zoom, and images for phloem unloading assay and meristem growth measurements were scanned under 20× objective. Excitation and emission wavelength were, respectively, 405 nm and 420–470 nm for calcofluor, 488 nm and 489–505 nm for GFP, and 561 nm and 600–650 nm for propidium iodide.

## Data Availability

Not applicable.

## Supplementary Materials

The following supporting information can be downloaded at: https://www.mdpi.com/article/10.3390/ijms23042063/s1.
