# Supplementary material for: Heat Stress Reduces Root Meristem Size via Induction of Plasmodesmal Callose Accumulation Inhibiting Phloem Unloading in Arabidopsis
Source: Int J Mol Sci. 2022 Feb 13;23(4):2063. doi: 10.3390/ijms23042063 (PMC8879574; doi:10.3390/ijms23042063)
Supplement: Supplementary file 1 [file ijms-23-02063-s001.zip › ijms-1581008-supplementary.pdf]

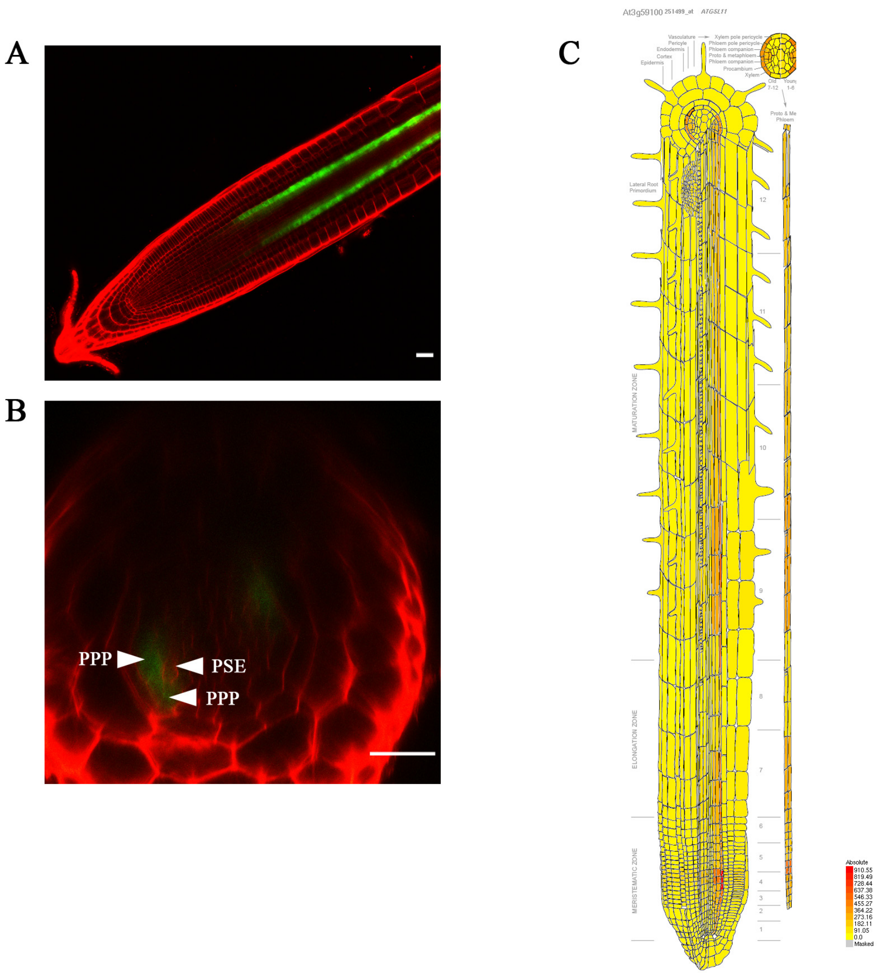

Figure S1: CalS8 and CalS6 expression are enrichment in the PPP cells.

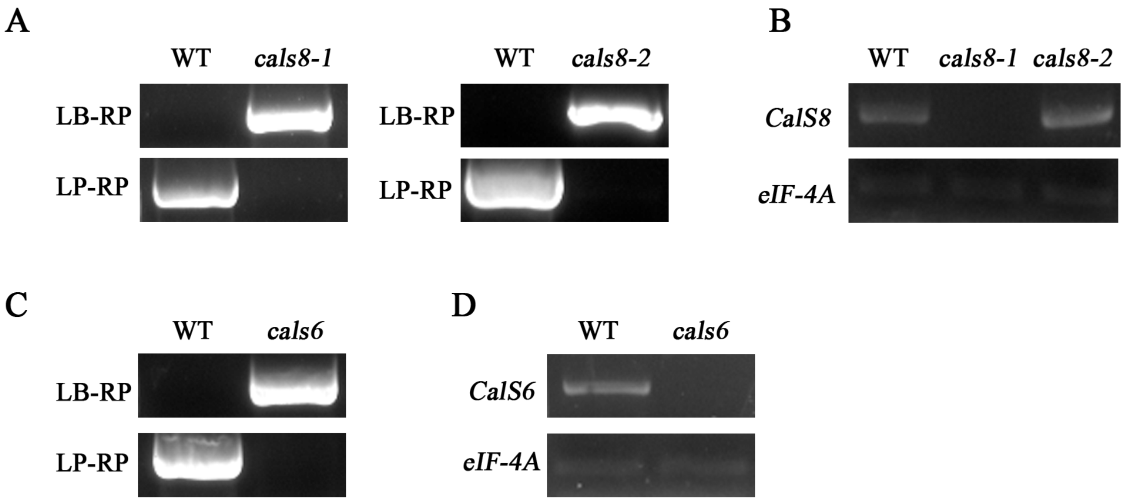

Figure S2: Selection and confirmation of knock-out mutations in cals8-1, cals8-2 and cals6.

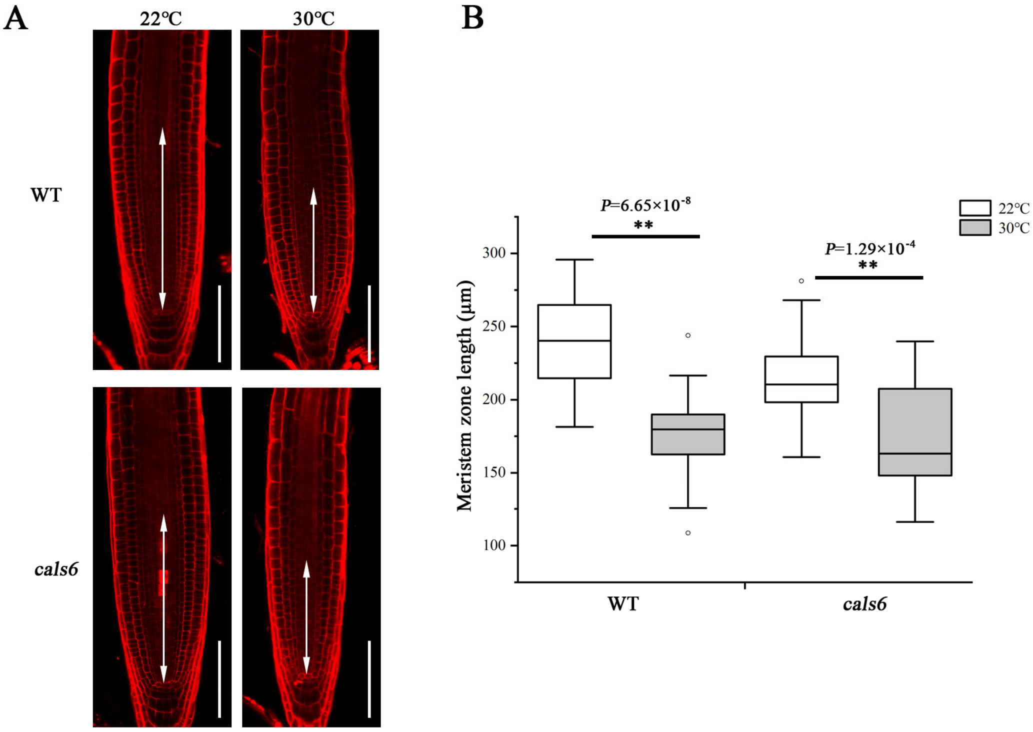

Figure S3: Loss of CalS6 does not rescue the meristem growth inhibition by heat stress.
